# From the prion-like propagation hypothesis to therapeutic strategies of anti-tau immunotherapy

**DOI:** 10.1007/s00401-019-02087-9

**Published:** 2019-11-04

**Authors:** Morvane Colin, Simon Dujardin, Susanna Schraen-Maschke, Guy Meno-Tetang, Charles Duyckaerts, Jean-Philippe Courade, Luc Buée

**Affiliations:** 1 Univ. Lille, Inserm, CHU-Lille, Lille Neuroscience and Cognition, Place de Verdun, 59045 Lille, France; 2 Mass General Institute for Neurodegenerative Disease, Massachusetts General Hospital, Harvard Medical School, Charlestown, MA USA; 3 UCB Celltech, 208 Bath Rd, Slough, SL1 3WE UK; 4 Department of Neuropathology, Sorbonne Université, Assistance Publique Hôpitaux de Paris, Inserm, CNRS, Institut du Cerveau Et de La Moelle, Hôpital Pitié-Salpêtrière, 75013 Paris, France; 5 UCB Biopharma, Chemin du Foriest, 1420 Braine l’Alleud, Belgium; 6 Inserm UMR-S 1172, ‘Alzheimer and Tauopathies’, Bâtiment Biserte, rue Polonovski, 59045 Lille Cedex, France

**Keywords:** Immunotherapy, Alzheimer’s disease, Seeding, Progressive supranuclear palsy, Secretion, CSF, Plasma, Tau

## Abstract

The term “propagon” is used to define proteins that may transmit misfolding in vitro, in tissues or in organisms. Among propagons, misfolded tau is thought to be involved in the pathogenic mechanisms of various “tauopathies” that include Alzheimer's disease, progressive supranuclear palsy, and argyrophilic grain disease. Here, we review the available data in the literature and point out how the prion-like tau propagation has been extended from Alzheimer's disease to tauopathies. First, in Alzheimer’s disease, the progression of tau aggregation follows stereotypical anatomical stages which may be considered as spreading. The mechanisms of the propagation are now subject to intensive and controversial research. It has been shown that tau may be secreted in the interstitial fluid in an active manner as reflected by high and constant concentration of extracellular tau during Alzheimer’s pathology. Animal and cell models have been devised to mimic tau seeding and propagation, and despite their limitations, they have further supported to the prion-like propagation hypothesis. Finally, such new ways of thinking have led to different therapeutic strategies in anti-tau immunotherapy among tauopathies and have stimulated new clinical trials. However, it appears that the prion-like propagation hypothesis mainly relies on data obtained in Alzheimer’s disease. From this review, it appears that further studies are needed (1) to characterize extracellular tau species, (2) to find the right pathological tau species to target, (3) to follow in vivo tau pathology by brain imaging and biomarkers and (4) to interpret current clinical trial results aimed at reducing the progression of these pathologies. Such inputs will be essential to have a comprehensive view of these promising therapeutic strategies in tauopathies.

## Introduction

Alzheimer's disease (AD) is a genuine challenge for the pharmaceutical industry. The current drugs on the market show only low efficacy, and the clinical trials of the last 20 years have all been negative in phase 3. With the hegemony of the amyloid cascade hypothesis and the focus on amyloid precursor protein (APP) and its metabolites (Aβ and amyloid precursor protein intracellular domain: AICD), therapeutic strategies targeting tau protein, a major component of neurofibrillary tangles and neuropil threads, have emerged only in recent years. However, tau aggregates not only in AD, but also in many other highly heterogeneous pathologies called tauopathies. Understanding tau and tauopathies is essential before designing a therapeutic approach. Tau may be an excellent therapeutic target, but should the strategy be similar among tauopathies? The hypothesis of the amyloid cascade has oriented most of the research towards APP and Aβ; in the tau field, the hypothesis of a prion-like propagation has similarly captured the research effort and oriented the new therapeutic approaches such as immunotherapy into new directions.

In this review, we combined the data obtained in humans and those generated from experimental models in the context of the prion-like propagation hypothesis for tauopathies. We evaluated the current knowledge of tau biology in intra- and extracellular compartments (brain and peripheral fluids), described recent breakthroughs, and highlighted some unanswered questions. Finally, with this knowledge, we wondered whether we could predict the mode of action and the target engagement of immunotherapy approaches among tauopathies.

## Tau isoforms and inclusions

**Fig. 1:**
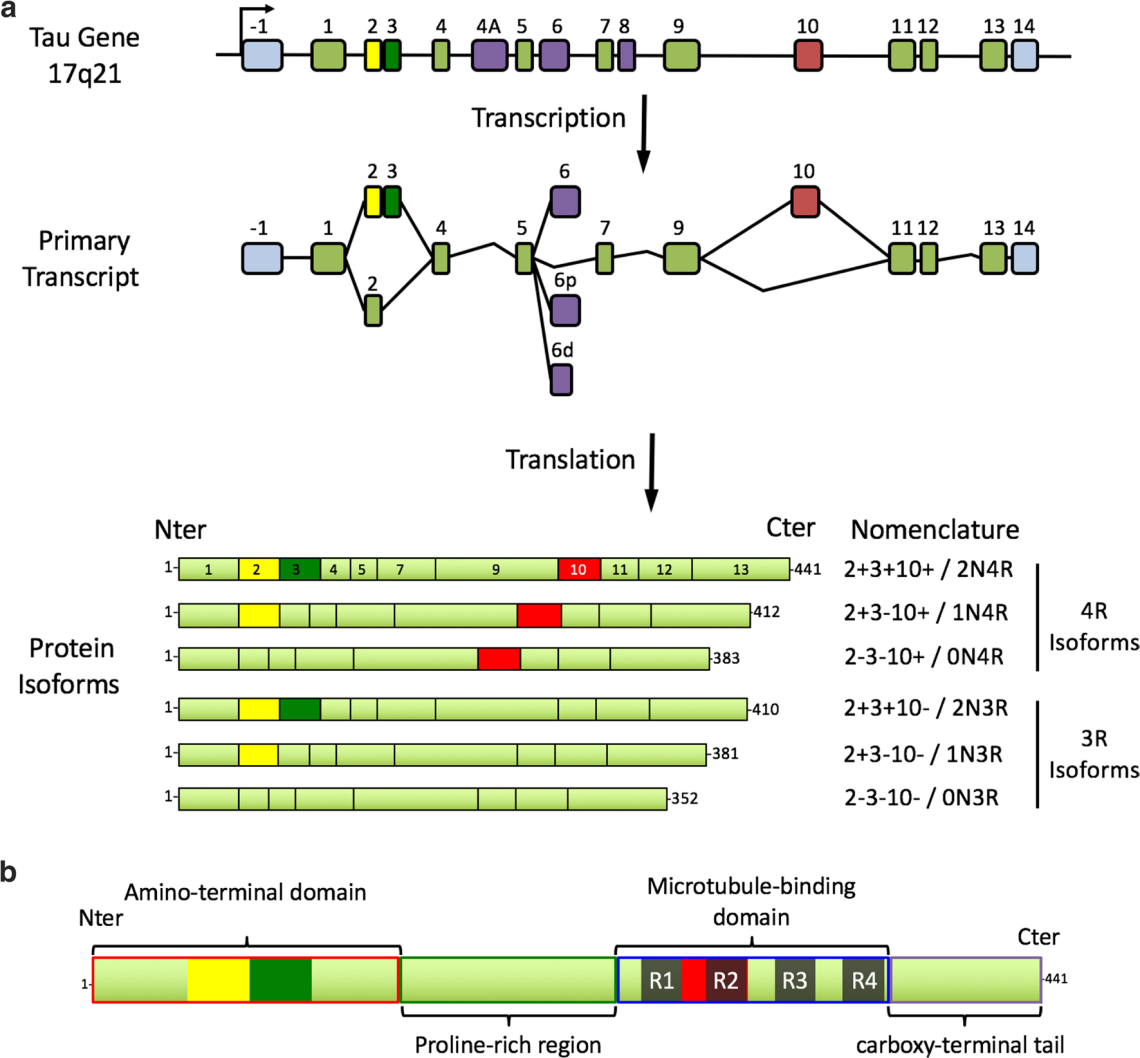
**a** Schematic presentation of the MAPT gene, its primary transcript and the six protein isoforms expressed in the human brain. The MAPT gene is composed of 16 exons. In the brain, exons 4A and 8 are excluded from the primary transcript. Exons 1, 4, 5, 7, 9, 11, 12 and 13 are constitutive, whereas exons 2, 3, 6 and 10 are alternative. Exon 3 never appears independently of exon 2. Exons 1 and 14 are present in the mRNA, but are never translated. Six main transcripts are present in the adult brain: 2 – 3 – 10 − or 0N3R; 2 + 3 − 10 − or 1N3R; 2 + 3 + 10 − or 2N3R; 2 – 3 − 10 + or 0N4R; 2 + 3 − 10 + or 1N4R; 2 + 3 + 10 + or 2N4R. **b** Tau structure. Four domains with different biochemical properties can be retrieved in tau protein: an acidic amino terminal region (corresponding to the expression of exons 1–5), a proline-rich domain (corresponding to the expression of exons 7 and 9), the MTBR with four repeated sequences (R1–R4), and a carboxy-terminal tail (exon 13). Modified from [28]

The advance of immunohistochemistry has revealed that approximately 20 neurodegenerative diseases, named tauopathies, are characterized by the accumulation of hyperphosphorylated tau (pTau) [165]. Tau is encoded by a single gene (*MAPT*) located on chromosome 17. The *MAPT* gene is mostly expressed in neurons [27], and due to alternative splicing of exons 2, 3 and 10, six main isoforms are found in the adult brain: 2 – 3 − 10− (0N3R), 2 + 3 − 10− (1N3R), 2 + 3 + 10− (2N3R), 2 − 3 − 10 + (0N4R), 2 + 3 − 10 + (1N4R), and 2 + 3 + 10 + (2N4R) [5, 81] (Fig. 1a).

Tau protein has four domains with unique biochemical characteristics and specific functions: (i) an acidic amino-terminal domain, (ii) a proline-rich region followed by (iii) microtubule-binding regions (MTBR) and (iv) a carboxy-terminal tail. The microtubule-binding regions contain three or four repeat domains (depending on the inclusion of exon 10) [38, 82, 90, 114] (Fig. 1b). Several post-translational modifications (PTMs) have been described on tau proteins [87]; phosphorylation dynamically regulates the physiological functions of tau [133, 160]. In tauopathies, tau is excessively and abnormally phosphorylated [10]. Other PTMs (acetylation, glycation, glycosylation, methylation, SUMOylation, truncation, ubiquitinylation, etc.) have also been described; some of them, such as acetylation, glycosylation and truncation, may also be related to the pathology and are considered as therapeutic targets [100].

**Fig. 2:**
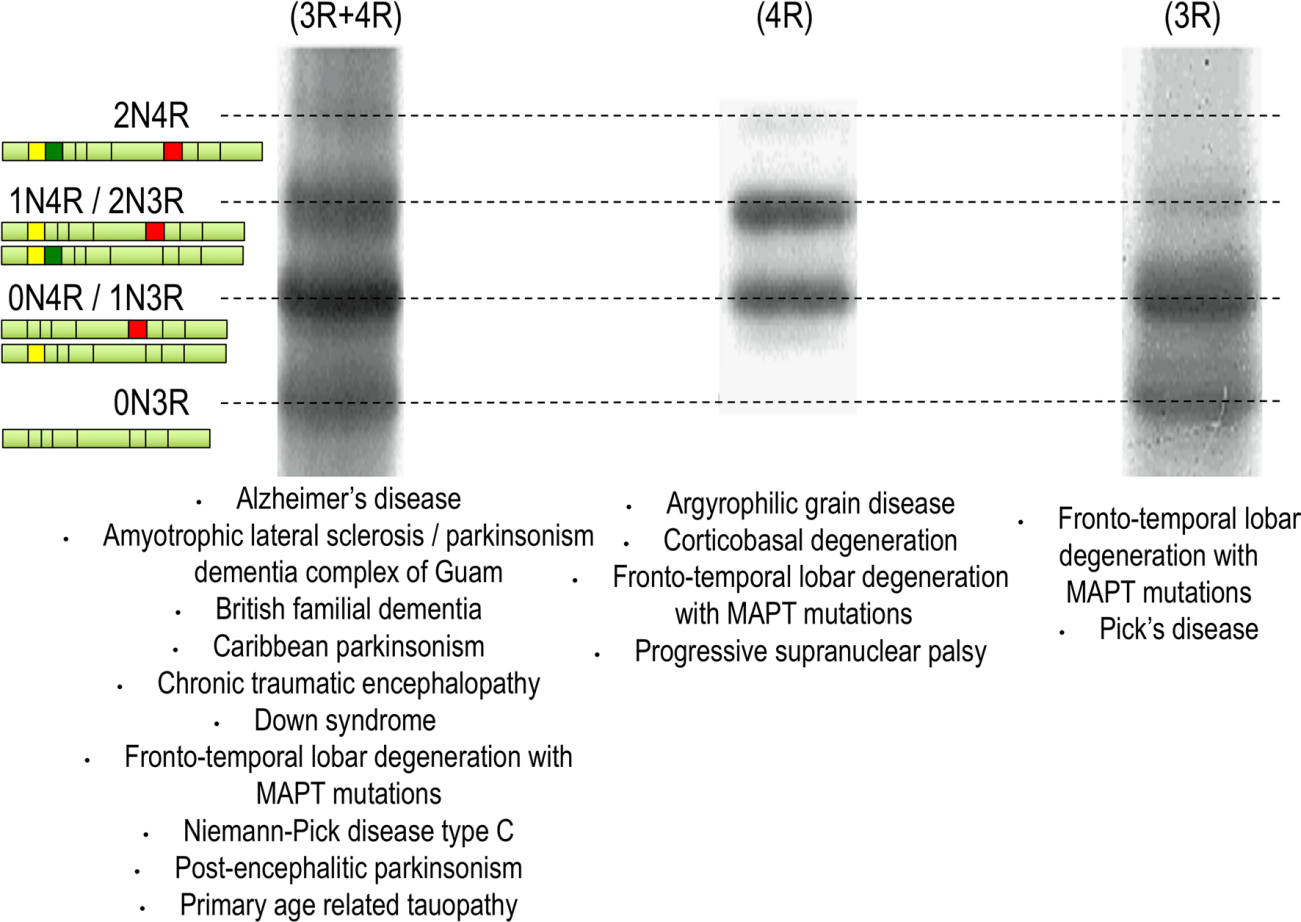
Tauopathy ‘barcode’. Western blots showing the electrophoretic profile observed with tau protein aggregates from patients with different tauopathies. In AD-like, the six tau isoforms are present in the aggregates. In PSP-like, only the 4R-tau proteins are aggregated. In Pick’s disease, only the 3R isoforms are aggregating. Modified from [160]

pTau may accumulate in the cell bodies of neurons without forming fibrillary aggregates—a change called a “pre-tangle”. Moreover, pTau may aggregate in the cell bodies of neurons (neurofibrillary tangles = NFTs) or in the cell processes (neuropil threads = NT), and NT may be axonal (as in the corona of the senile plaque) or dendritic. Under electron microscopy, tau aggregates are principally made of paired helical filaments (PHF) in AD (3R and 4R), which is also the case in ‘primary age related tauopathy’ (PART) in chronic traumatic encephalopathy (CTE) and in some less-common disorders (see Fig. 2). In progressive supranuclear palsy (PSP) and cortico-basal degeneration (CBD), tau aggregates are found both in neurons and glia and are made of 4R straight tau filaments. Other specific neuronal tau inclusions are Pick bodies (seen in Pick disease), in which 3R tau aggregates in the neuronal cell body adopt a spherical shape, and argyrophilic grains made of 4R tau (seen in argyrophilic grain disease (AGD)) are located in presynaptic terminals. The glial inclusions may involve astrocytes: in astrocytic tufts, suggestive of PSP, all processes of the astrocyte are filled with pTau; in astrocytic plaques, seen in CBD, pTau immunoreactivity is found at the end of the astrocytic processes [108, 110]; and they may involve oligodendrocytes where they form coiled bodies (abundant in PSP, CBD and AGD). The ratio between 3 and 4R tau explains why specific sets of migration bands have been recognized by Western blotting (Fig. 2). Recent structural studies by cryo-electron microscopy have confirmed the presence of different tau structures among AD, Pick’s disease and CTE [67, 68, 71].

Genetic frontotemporal lobar degeneration (FTLD-MAPT formerly called FTDP-17, [74]) is of particular interest. It is caused by autosomal dominant *MAPT* mutations mainly located in the sequence regulating exon 10 alternative splicing or encoding microtubule-binding regions. Most coding region mutations often act on both nucleation and fibrillogenesis [12, 36]. They are widely used for studying the pathological mechanisms of tauopathies, especially in animal models [61].

**Fig. 3:**
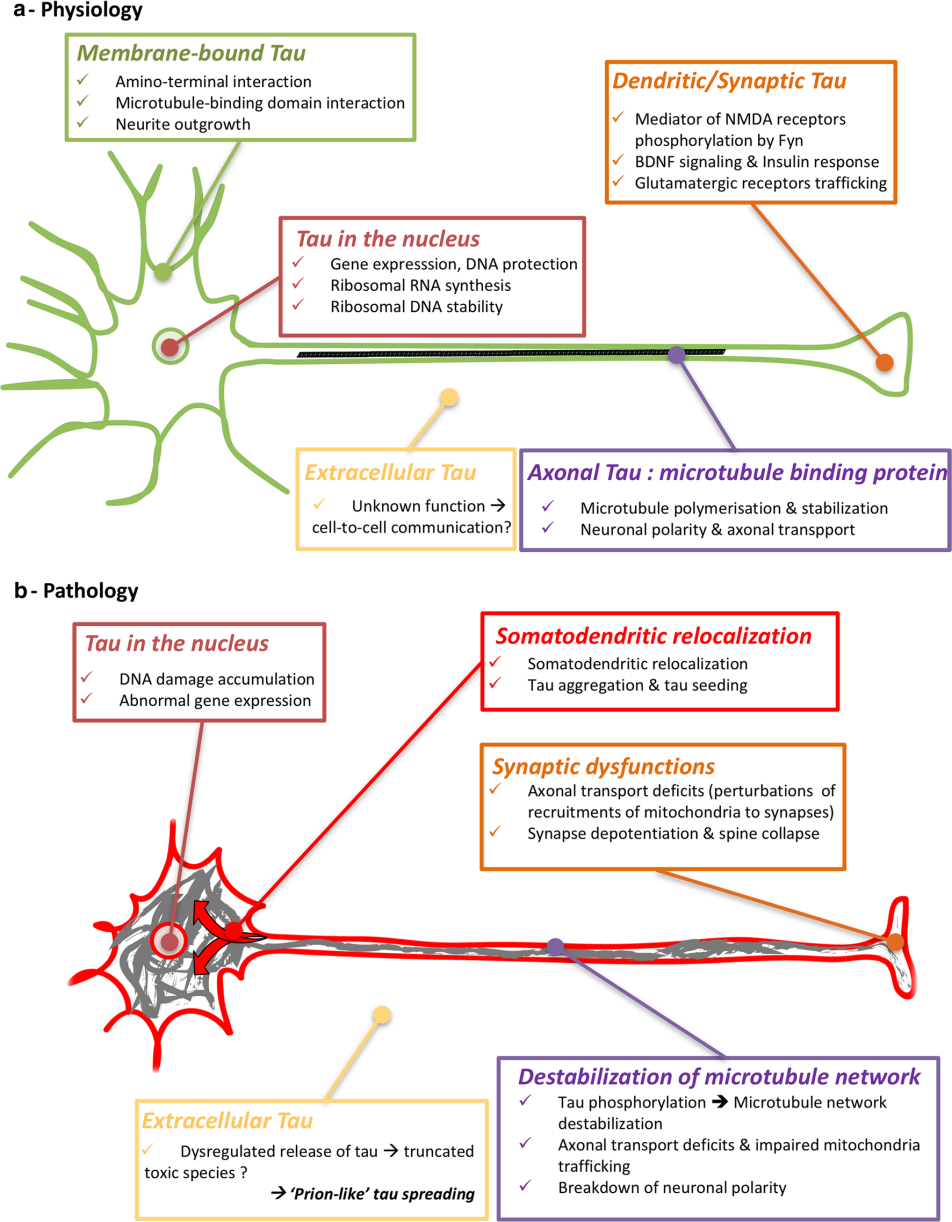
Functions and dysfunctions of tau proteins **a** Physiologically, tau protein is mainly located in the cytoplasm of axons to stabilize the microtubules. Other minor locations of tau can be observed, such as in the nucleus [117], bound to the membranes [23] and in dendrites [99]. These locations are associated with atypical functions of tau [164], such as structuring chromatin and protecting nucleic acids from oxidative stress [15, 39, 120, 144, 169, 182, 183], insulin signalling by binding to PTEN in the somato-dendritic compartment [121], mediating neuronal activity via the Fyn kinase and NMDA receptors in dendrites [29, 99, 129]. Tau proteins are also retrieved in extracellular fluids. **b** During tauopathies, tau proteins are excessively and abnormally phosphorylated and then aggregate, leading to a substantial loss of function. In particular, the microtubule network is destabilized, tau proteins are relocalized and synaptic deficits appear. Extracellular tau proteins are modified, and their functions are not completely understood even if they could participate in tau pathology propagation in the brain

Altogether, tauopathies are the consequence of several molecular dysfunctions likely due to PTM and protein-folding deregulations. These deregulations are associated with the development of conformational changes, oligomerization and finally, intracytoplasmic aggregation of tau. All these processes have a significant impact on cell physiology and particularly on tau functions [164] (Fig. 3). Neuropathology, however, shows the large variety of cerebral areas and cell populations (glial or neuronal) affected; it also indicates that the type of tau aggregation is diverse, as shown by the multiplicity of tau inclusions and their molecular composition.

## Prion-like propagation hypothesis

Tauopathies and other neurodegenerative disorders are reminiscent of prion diseases. The latter are indeed related to a pathological change (misfolding) in the molecular conformation of the normally present prion protein and to the capacity of misfolded prions to induce misfolding by contact with a normally folded prion. Interestingly, different strains of prion exist with dissimilar fibre conformations that are transmissible [175]. There are at least two steps in the development of prion diseases: (1) misfolding induction and (2) transport of the misfolded prion that permits contact with still normal prions. Step 1 is called seeding, and step 2 is propagation. Propagation can take place by diffusion in the extracellular space or along the axonal paths.

Seeding and propagation have been reported not only with prions, but also with Aβ [102] and alpha-synuclein [97]. The term “prion-like” has been attributed to this molecular behaviour when applied to proteins other than prions; such proteins have been called “propagons” and may induce protein misfolding in test-tubes, tissues, organisms or between subjects (infectious). Although there is no clear evidence of such interindividual transmission for tau, in several tauopathies, tau pathology progresses by well-defined “stages”, which may support prion-like propagation. Here, the term “prion-like propagation hypothesis” includes the two steps: seeding and propagation.

### Pathology stages and the “propagation hypothesis” in humans

**Fig. 4:**
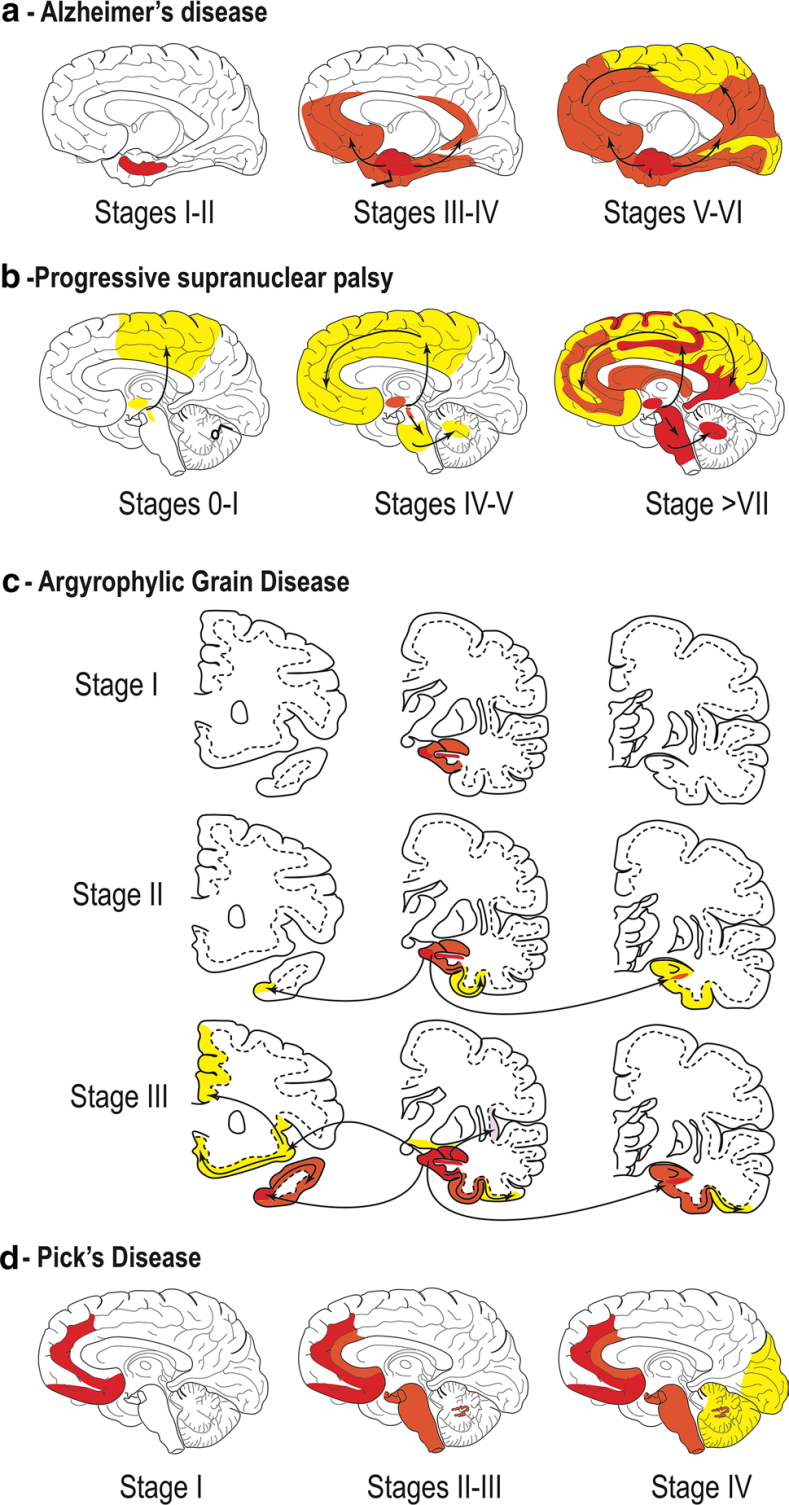
**a** Staging of tau pathology in AD. Topographic distribution of tau lesions at the different stages of tau pathology in schemes of brains in medial views. Stages I and II, tau lesions invade entorhinal and transentorhinal regions. Stages III and IV: lesions involve the associative areas of the neocortex, and finally, during stages V and VI, tau lesions invade all the primary and secondary neocortical areas. From [22]. **b** Staging of tau pathology in PSP. Topographical distribution of tau lesions at the different neuropathological stages of PSP in schematic brain representations in medial views. Stages 0/I—Only the pallido-luyso-nigral complex shows tau pathology with weak involvement of the premotor cortex. Stage II/III—Tau pathology reaches the basal ganglia, pedunculopontine nucleus and dentate nucleus. Stages IV/V—Frontal and temporal lobes are involved. Stages VI/VII—Subthalamic nucleus, substantia nigra, internal globus pallidus, neocortical areas, pedunculopontine nucleus and cerebellum are more severely affected. Modified from [189]. **c** Staging of tau pathology in AGD. Topographical distribution of argyrophilic grains at the different stages of tau pathology evolution in three coronal sections. Stage I—argyrophilic grains are located in the ambiens gyrus, anterior CA1, anterior entorhinal area and amygdala. The stage II—medial temporal lobe is more affected by the involvement of the posterior subiculum, entorhinal and transentorhinal cortices. Stage III grains invade the anterior cingulate gyrus, septum, accumbens nucleus, rectus gyrus, insular cortex and hypothalamus. Modified from [152]. **d** Staging of tau pathology in Pick’s disease (PiD). Topographical distribution of Pick bodies at the different stages of tau pathology evolution in schematic brain representations in medial views. Stage I Tau pathology is deposited in the limbic and neocortical frontotemporal regions as well as the angular gyrus. Stages II/III—White matter tracts, subcortical structures, serotonergic/noradrenergic brainstem nuclei are affected, followed by the primary motor cortex and pre-cerebellar nuclei. Finally, in stage IV, tau is deposited in the visual cortex as well as in the cerebellar granular layer and brainstem white matter. Modified from [96]

In AD, tauopathy is found sequentially in the transentorhinal–entorhinal areas, hippocampus and limbic areas and finally in the associative and then primary neocortical areas [20] (Fig. 4a). In PSP, the tauopathy is initially confined to the pallido-luyso-nigral system and then involves the basal ganglia, the pontine nuclei and the dentate nucleus, and finally the frontal and parietal lobes—with not only the extent but also the severity of the involvement increasing with time [181, 189] (Fig. 4b). In AGD, the tauopathy is initially limited to the ambient gyrus and its vicinity; it involves secondarily the temporal pole, the subiculum and the entorhinal cortex and finally, the septum, insular cortex, and anterior cingulate gyrus [152] (Fig. 4c). In Pick disease, Pick bodies are initially seen in limbic regions; subcortical regions (the thalamus, striatum, locus coeruleus, raphe nuclei, dorsal motor nucleus of the vagus nerve, and reticular formation) as well as the primary sensory cortex are affected secondarily, followed by the primary motor cortex, the inferior olivary nucleus, and finally, the primary visual cortex [96] (Fig. 4d).

The active cell-to-cell transfer of tau has not yet been visualized in the human brain, even if some positron emission tomography (PET) and magnetic resonance imaging (MRI) studies suggest that it may occur. Similar to the Braak stages, there are strong arguments to support a hierarchical pathway of neurodegeneration along neural networks in AD [42, 48, 91].

The progression by stages in those tauopathies has suggested that two mechanisms, not necessarily antagonists, could be involved: progression of a pathogenic molecule along neuronal connections or selective vulnerability (pathoclisis). Given the complexity of the neuroanatomy of the central nervous system, the existence of connections between two regions of the brain that are successively involved is a necessary condition, but not proof, of the progression along neuronal connections. On the other hand, the presence of tau pathology in astroglial cells cannot be directly explained by the connections. In fact, in PSP, at transcriptomic level, tufted astrocytes appear to be associated with a microglial gene-enriched immune network whereas neurofibrillary tangles would be linked to a brain co-expression network enriched for synaptic and PSP candidate risk genes [3]. Numerous uncertainties remain concerning the progression of the tauopathies and neurodegenerative diseases in general, which explains the current controversies [65, 113, 177].

Notably, the diversity of the tauopathies likely implies a diversity of pathogenic mechanisms. It appears implausible that one single treatment could apply to all of them. Conversely, a progression through connections like in AD would be compatible with a prion-like mechanism and thus it is fair to explore this hypothesis among tauopathies.

The most striking evidence of transmission of the pathology through connection is found in AD. Connectivity is explicitly implicated by Braak and Braak in the description of their stages [20]. In addition, an interesting autopsied AD case has also been reported showing that a 27-year meningioma removal by neurosurgery had massively disconnected a piece of cortex. A local dissociation between neuritic and Aβ pathologies was seen. Neuropathological analyses fully confirmed AD diagnosis with numerous amyloid deposts and NFTs. However, in the disconnected cortical brain region where amyloid deposits were numerous, no neurofibrillary tangle was observed suggesting that their presence is determined by the neural connections [64].

However, other hypotheses have been proposed. According to one of them, AD would be a phylogenic neurodegenerative disease of higher primates following an evolutionary change in the primate genome [145]. According to another hypothesis, the pattern of neurodegeneration may be explained by the possible existence of chemically defined neuronal subpopulations that are highly vulnerable in AD [92]. In fact, this neurodegeneration pathway bears a striking resemblance to the inverse sequence of cortical myelination [19].

Recently, the hypothesis of prion-like propagation has offered an explanation for this hierarchical pathway of neurodegeneration [21]. The relevance of the widening of this concept is still being discussed. There is still controversy about tau propagation in CBD, Pick’s disease and PSP [24, 48, 59, 73, 96, 137, 181, 189]. The recent use of tau PET imaging will be helpful in bringing new insights into such debate [112].

### Human tau in peripheral fluids: a new argument for the prion-like propagation hypothesis?

The propagation, included in the prion-like hypothesis, implies tau passage in the extracellular space. The passage of misfolded tau in the extracellular space could be passive and related to neuronal death. Currently, there is evidence that misfolded tau is actually secreted [194, 195]. As a consequence of the secretion step, tau is found in extracellular fluids (in a vesicular or non-vesicular form) [130, 140]. The interstitial fluid (ISF) that is found in the brain between the cells may be directly sampled and analysed by microdialysis. In humans, microdialysis has been used in cases of acute brain trauma to monitor brain metabolism [89], but the technique cannot be applied to neurodegenerative diseases such as tauopathies. Approximately, 20% of the cerebrospinal fluid (CSF) originates from ISF [157]; changes in ISF protein concentrations are thus probably reflected by changes in the CSF. Tau proteins originating mainly from neurons have decreasing concentrations from ventricles (320 pg/mL) to lumbar subarachnoid space (210 pg/mL) [147]. This observation suggests that CSF tau originates mainly from ISF and CSF is the extracellular compartment closest to the brain, which is most accessible for sampling. CSF tau has been extensively studied to investigate the pathophysiological processes occurring in neurodegenerative diseases in living patients. The presence of tau in CSF was identified by electrophoresis in the mid-80s [37, 77] and quantified from 1993 by sandwich enzyme-linked immunosorbent assay [180]. Human CSF contains tau levels typically ranging from approximately 100 to 1200 pg/mL [123]. Recently, CSF tau kinetics were quantified by stable isotope labelling kinetics (SILK) labelling in healthy individuals (half-life = 23 ± 6.4 days, tau production rate of 26.3 pg/mL/day ± 9.2 and CSF level of 812.7 ± 186 pg/mL) [158]. Regarding CSF tau quantification in tauopathies, only individuals with AD clearly display increased tau concentrations in CSF [14].

Until very recently, tau could not be detected in the plasma, because of its very low concentration compared with that in CSF. Although protein transfer from ISF and CSF to blood is very low, and its mechanisms are not well known (efflux across the blood–brain barrier [11], along the glymphatic paravascular pathway [95], clearance through the dural lymphatic system in blood [139]). Tau levels in plasma might thus also be altered by changes in the brain [11] (Table 1).

**Table 1:** Plasma tau levels by diagnostic group in AD/MCI (mild cognitive impairment) and FTD (frontotemporal degeneration) studies

Study	Group 1 (N/age) PlTau (pg/mL) Mean/SD	Group 2 (N/age) PlTau (pg/mL) Mean/SD	Group 3 (N/age) PlTau (pg/mL) Mean/SD	Method	Comparison of plTau according to diagnostic group	Correlation of plTau with other biomarkers	Association of plTau with disease hallmarks
[200]	**AD** (54/75 years) **T: 8.80 (10.1)**	**MCI** (75/68 years) **T: 4.68 (4.25)**	**Ctrls** (25/74 years) **T: 4.43 (2.83)**	S	*AD > MCI and ctrls *MCI-AD = stable MCI	With CSF Tau: No	–
[124]	ADNI **AD** (179, 75 years) **T: 3.12 (1.50)**	ADNI **MCI** (195, 75 years) **T: 2.71 (1.32)**	ADNI **Ctrls** (189, 76 years) **T: 2.58 (1.19)**	S	*AD > MCI and ctrls *MCI = Ctrls *MCI-AD = Ctrls	*With CSF Tau or pTau: No *With CSF Aβ42: yes	*With worse cognition (MMSE, ADAScog) *With more atrophy (hippocampal/ventricular volume L) *With hypometabolism (FDG-PET)
	BioFINDER **AD** (61, 76 years) **T: 5.37 (2.56)**	BioFINDER **MCI** (212, 71 years) **T: 5.46 (2.71)**	BioFINDER Ctrls (274, 73 years) **T: 5.58 (2.51)** BioFINDER SCD (174, 70 years) **T: 5.21 (2.72)**		No difference	*With CSF Tau and pTau: only in AD group	–
[51]	All patients: (539/80 years)	MAYO **MCI** (161/–) **T: 4.34**	MAYO **ctrls** (378/–) **T: 4.14**	S	MCI = Ctrls		*With worse memory performance *With abnormal cortical thickness
[131]	/	DELCODE **SCD** (111, 71 years) **T: 3.4 (1.2)**	DELCODE **Ctrls** (134, 68 years) **T: 3.6 (1.7)**	S	MCI = SCD	*With CSF pTau, plTau or Aβ42: No	–
[56]	ADNI **AD** (168, 75 years) **T: 3.13 (1.3)**	ADNI **MCI** (174, 74 years) **T: 2.81 (1.2)**	ADNI **Ctrls** (166, 75 years) **T: 2.71 (1)**	S	–	–	*With cortical thickness: NO *With grey matter density in medial temporal lobe
[126]		MAYO **MCI** (123/80 years) **T: 4.5 (1.8)**	MAYO **ctrls** (335/81 years) **T: 4.2 (1.5)**	S	High tT *Among Ctrls: association with increased risk of MCI *Among MCI: No association with increased risk of dementia	–	*Association with cognitive decline at 15 months follow-up: Among Ctrls: No among MCI: yes (visuospatial ability, global cognition)
[174]	**AD** (20, 77 years) **pT: 0.17 (0.16)**		**Ctrls** (15, 76 years) **pT: 0.04 (0.07)**	S	pT: AD > Ctrls Cut-off = 0.092 AUC = 0.786	–	–
[72]	**bvFTLD** (71, 64 years) **T: 1.96 (1.07)**	**PPA** (83, 67 years) **T: 2.65 (2.15)**	Ctrls (22, 69 years) **T: 1.67 (0.50)**	S	*bvFLTD and PPA > ctrls	*Serum NfL: No	*Brain volume: no *Disease duration: no
	**Genetic subgroups:** *MAPT *(12): **T: 2.62 (1.39)** *GRN* (9): **T: 2.22 (1.60)** *C9ORF* (15): **T: 1.93 (0.70)**				*MAPT > *GRN* and *C9ORF*		
[127]	MAYO **AD** (40, 68 years) **T: 7.2 (2.8)** **pT: 11.6 (4.1)**	**MAYO MCI** (57, 71 years) **T: 5.9 (2.8)** **pT: 9.0 (13.9)**	**MAYO Ctrls** (172, 72 years) **T: 5.9 (61.9)** **pT: 6.4 (6.4)**	S M	T: AD > MCI = Ctrls pT: AD > Ctrls	–	Higher plT associated with Aβ PET and Tau PET
[40]	**AD** Discovery cohort (25, 61 years) Validation cohort (23, 72 years)	**MCI-AD** Discovery cohort (21, 65 years) **NtT: 4.85** Validation cohort (22, 73 years) **NtT: 3.42**	**Ctrls** Discovery cohort (9/10, 60/70 years) **NtT: 5.12** Validation cohort (41, 72 years) **NtT: 3.40**	S	NtT separate ctrls from discovery cohort/validation cohort: MCI-AD (AUC = 0.88/0.79) AD (AUC = 0.96/0.75)	–	–
[138]	FHS **Incident dementia** (134, –) **Incident AD** subgroup (105, –)	**Autopsy sample subgroup** (42, 82 years) **Confirmed AD** (11, –)	FHS **Ctrls** (1319, 75 years)	S	T > median associated with: *Greater risk of dementia (HR = 1.62) *Greater risk of AD (HR = 1.76)		*Poorer cognitive performance *Smaller hippocampal volume *Higher burden of NFT in the medial temporal lobe (autopsy subgroup)
[41]		**Middle-aged Ctrls** (56, 58 years) **T: 14.3 (4.9)**	**Old Ctrls** (70, 74 years) **T: 18.1 (7.3)**	I	*Old ctrl > middle-aged ctrl *Age was positively associated with tT	–	*Volume of subcortical brain structures: No *Thickness of cortical regions: No
[118]	BSHRI **AD** (16, 82 years) **T: 34.5 (3.8)** NTUH AD (31, 72 years) **T: 52.5 (2.7)**	–	BSHRI **Ctrls** (16, 82.5 years) **T: 20.5 (1.2)** NTUH Ctrls (61, 64 years) **T: 14.0 (1.9)**	I	AD > Ctrls (*p* < 0.02) Cut-off: 25 pg/mL AUC = 0.97, Se = 89%, spe = 94%	*Combined with plAβ42: AUC = 0.98, Se = 94%, spe = 92%	–
[196]	Mild **AD** **T: 37.5 (12.3)** **pT: 6.1 (1.6)**	**MCI due to AD** **T: 33.0 (10.2)** **pT: 4.4 (1.8)**	**Ctrls** **T: 18.8 (10.2)** **pT: 2.5 (1.1)**	I	T: MCI-AD > Ctrls pT: AD > MCI-D > Ctrls	–	–
[197]	**AD** (29, 72 years) **T: 55.4 (22.4)** **FTLD** (26, 62) **T: 41.3 (20)**	**MCI-AD** (24, 71 years) **T: 33.3 (7.8)**	**Ctrls** (66, 65 years) **T: 13.4 (7.8)**	I	AD, MCI-AD and FTD > Ctrls cut-off: 17.4 pg/mL AUC = 1.0 (for AD and MCI-AD), AUC = 0.96 (for FTLD)	–	–
[115]	**PSP** (6, 67 years) **T: 18.9 (2.1)** **pT: 3.8 (0.7)** **CBD** (3, 62 years) ***T: 14.7 (1.0)*** ***pT: 3.2 (0.2)***	**FTLD** without Parkinsonism (25, 59 years) **T: 41.5 (1.1)** **pT: 6.8 (0.3)** **FTLD-Park** (6, 58 years) **T: 24.1 (2.0)** **pT: 6.8 (0.2)**	**Ctrls** (35, 63 years) **T: 12.1 (1.0)** **pT: 2.5 (1.1)**	I	*T and pT in all disease groups > ctrls *T FTLD > FTLD-Park	–	–

Column 1: study reference; columns 2, 3 and 4: patient groups and plTau levels in bold (T: total tau, pT: phospho tau Thr181, NtT: N-terminal fragment of tau); column 5: method used for plasma tau analysis (S: SIMOA (single molecule array technology); I: IMR (immunomagnetic reduction); M: MSD (Mesoscale Discovery); columns 6, 7, 8: results according to patient groups, other biomarkers and disease hallmarks, respectively

*PlTau* plasma tau, *pTau* phospho tau, *BSHRI* Banner Sun Health Institute (United States), *NTUH* National Taiwan University Hospital (Taiwan), *AUC* Area Under the Curve (ROC analysis), *ADNI* Alzheimer's Disease Neuroimaging Initiative (American Research Program on Alzheimer's Disease), *BioFINDER* Biomarkers For Identifying Neurodegenerative Disorders Early and Reliably (Swedish Study), *MAYO* Mayo Clinic (Rochester, Minnesota, USA), *DELCODE* DZNE-Longitudinal Cognitive Impairment and Dementia Study (DELCODE) conducted by the DZNE (German Center for Neurodegenerative Diseases), *FHS* Framigham Heart Study (US community-based cohort), *SCD* Subjective Cognitive Decline, *PPA* Primary Progressive Aphasia

Altogether, the prion-like propagation hypothesis is further supported by the increased and stable concentrations of extracellular tau in AD. In addition, recent work has shown that human CSF from AD patients is able to induce tau seeding in experimental models [163]. However, without a full characterization of tau species present in human ISF, it is hazardous to infer its concentration from that in CSF or in plasma. Animal models offer the possibility to recover ISF [192, 193].

## Modelling of the prion-like propagation

Data from humans allowed the development of experimental models to assess whether the ‘prion-like’ propagation hypothesis is implicated in tauopathies and especially whether extracellular tau is relevant to this hypothesis. These models were thus designed to investigate the prion paradigm in regard to human neuropathology. For instance, with the upregulation of Rab7A in AD patients [78–80], tau secretion seems to be regulated by this small GTPase involved in the trafficking of endosomes, autophagosomes, and lysosomes [149]. Similarly, tau secretion is correlated with Golgi dynamics [128], consistent with its fragmentation noted in AD [167]. This hypothesis involves a multi-step mechanism (tau secretion, uptake and subsequent seeding processes) that has been widely explored.

### Tau secretion

**Fig. 5:**
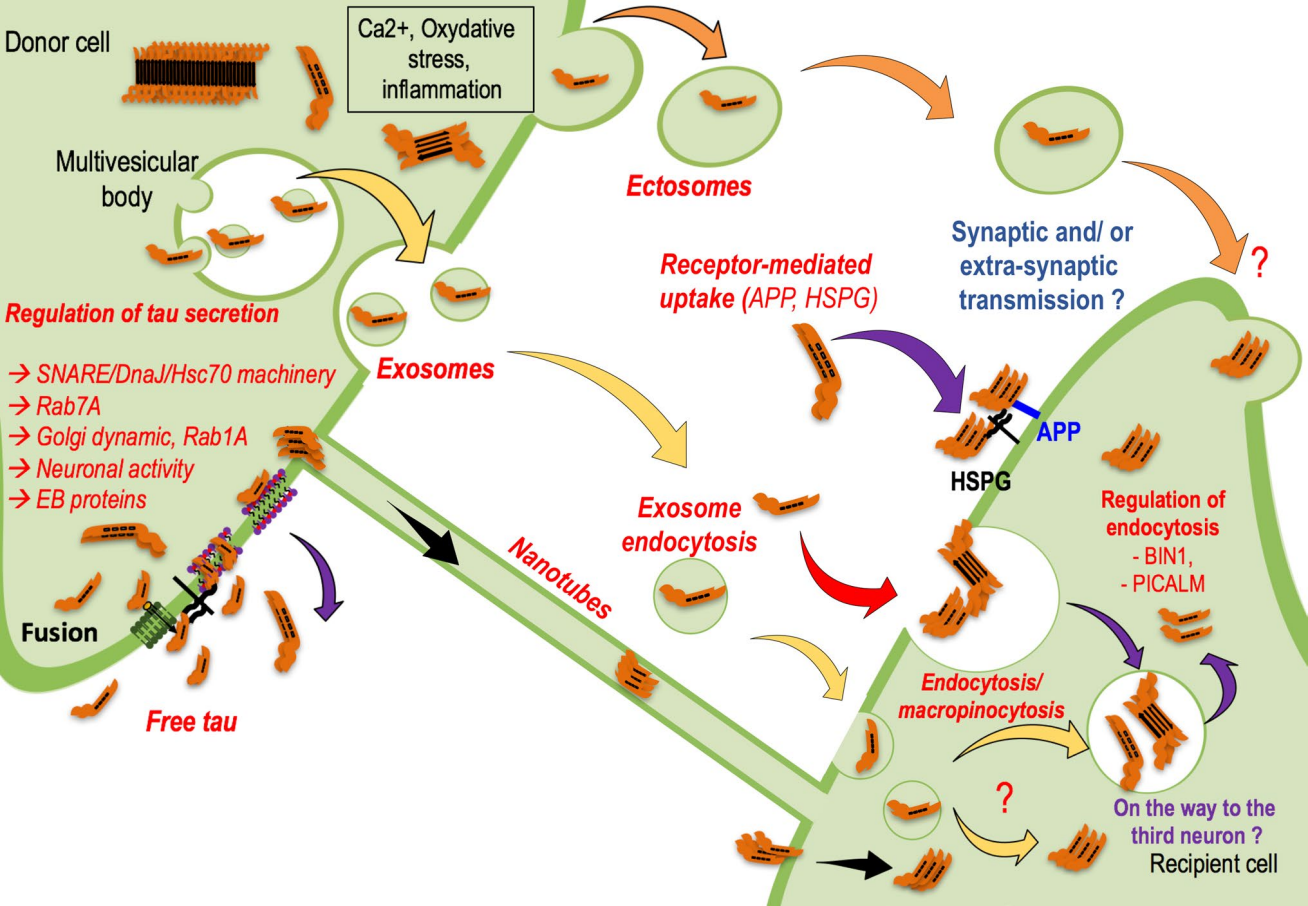
How is tau secreted and transferred into recipient cells? Tau secretion-Yellow-Tau protein could be carried by EVs, and the most investigated proteins are the exosomes, which are small vesicles (50–150 nm) coming from a subpopulation of intraluminal multivesicular bodies vesicles. Orange-Tau protein could also be carried by larger EVs named ectosomes (150–1000 nm) coming from the direct budding of the plasma membrane. Ectosome budding is regulated at least by calcium and oxidative stress, which are deregulated in many neurodegenerative disorders. Violet-Finally, tau protein is mainly found in a free form in extracellular fluids. How tau is secreted is not well documented, but a few papers are now investigating this mechanism and its regulation. Regardless of the shuttles and depending on the models used, tau has been identified in many forms in the extracellular compartment, and to date, no one has been able to decipher the toxic/propagative forms. Are those secreted species cleared from the interstitial fluid? Are they transferred to other brain cells to propagate the pathology? Is this information implied in normal brain cell-to-cell communications? Tau transfer-How is tau taken up and handled by the receiving cells? Whether tau transfer requires the synapse remains a matter of debate, and there is now some evidence that it might support the process. Nevertheless, the co-existence of the lateral transmission process should not be excluded. Black-Tau protein may move from cell to cell via nanotubes, membranous actin-rich structures that form between two cells inducing cytoplasmic and membrane exchanges. Yellow-to deliver tau, exosomes may be taken up by endocytosis in receiving cells. However, in this manner, the rest of the process is unclear. Is tau targeted to intracellular degradative compartments, such as lysosomes, to generate tau seeds that will in turn convert the non-pathological receiving cell into a pathological state? Is tau transferred to a third population due to the endosomal pathway? The exosomal transfer from the first to the third neurons via exosomes seems to be linked to the hijacking of secretory endosomes. The way ectosomes are taken up by receiving cells has not yet been investigated. Red-Tau proteins could also be internalized in the secondary neuron via an endocytosis mechanism. Such a process might be regulated by Bin1 and PICALM proteins, as both have been identified by GWAS as binding partners of tau

First, is it possible to identify a mechanism by which tau is found in peripheral fluids? The nature of secreted tau is debated in the literature [130, 141] (Fig. 5). Tau is secreted in a free form [60, 103, 125, 185], but it is also found inside nanotubes [1, 173] or associated with extracellular vesicles (EVs) [140]. Whereas nanotubes may be difficult to visualize in the human brain, secreted tau in EVs is found not only in experimental models but also in peripheral fluids (CSF [153, 185] and blood [70, 85, 190]) in AD patients. However, immunoassays in cell models revealed that whereas tau is mainly secreted in a free form in conditioned medium, only 10–20% of EVs contain tau [60, 185].

In this regard, tau is a cytosolic protein and does not follow the classical secretory pathway. It is translated into the cytosol, and the mechanisms modulating tau secretion are numerous and include tau oligomerization, tau truncation and mutations, tau interaction with plasma and organelle membranes and tau hyperphosphorylation [142]. For instance, non-conventional secretion of cytosolic proteins such as fibroblast-growth factor 2 (FGF-2) and interleukin 1β (IL1β) include oligomerization and truncation, respectively [119, 166]. Similar to FGF-2, soluble hyperphosphorylated tau oligomers may directly translocate across the plasma membrane upon binding to PI(4.5)P_2_, followed by retention on the cell surface through heparan sulfate proteoglycans (HSPG) [103, 125]. IL1ß secretion is also an interesting model for tau secretion. First, extrinsic factors, such as inflammation, are likely to modulate this non-conventional secretion. Additionally, a sequence containing human tau residues (amino acid 18 to amino acid 28) was recently shown to act as a binding motif for end binding proteins [159]. These proteins belong to the group of microtubule plus-end tracking proteins that have been implicated in the secretion of IL1β [184] and might also regulate tau secretion. The inflammasome may trigger a pathological cascade leading to enzyme activation, tau truncation and secretion. In human CSF, 99.9% of tau is truncated at the C-terminal domain, and major cleavage seems to occur around amino acids 210–230 [158]. This finding was confirmed by Cicognola and collaborators who described an important cleavage site at amino acid 224 [43]. In the presence of Aβ, such a mechanism may be exacerbated [158], supporting the prion-like propagation hypothesis in AD.

### Tau uptake/transfer

In the propagation hypothesis, secreted tau must be taken up by cells (Fig. 5). In vivo models recapitulating pathological propagation have been developed. Focal intraneuronal expression of tau was obtained using either a neuropsin promoter [55, 116, 187] or viral vectors [6, 30, 59, 60, 186]. Although both models show cell-to-cell transfer of tau, thorough controls are required to exclude transgene leakage [198], viral diffusion throughout the brain [63] or side-effects related to tau overexpression. Regarding this latter, the recent humanization of the murine MAPT gene clearly indicates that prion-like propagation occurs without overexpression [151]. Many other in vivo models investigating tau propagation are based on intracranial delivery of pathological material (recombinant oligomeric or fibrillar tau, human or mouse brain-derived material, etc.) [130] or peripheral administration [46] in transgenic and non-transgenic mice. These models bypass how tau is secreted from donor cells and focus on how tau is captured and lead to seeding in receiving cells. Some of these models suggest that over time, tau pathology appears in distant, synaptically connected areas, which suggests cell-to-cell propagation. However, the exact role of synapses in the tau propagation process remains under investigation, although seed competent tau was recently shown to be enriched in the synaptic fraction of AD brain-derived materials [57]. Nonetheless, we cannot exclude the possibility that the spread of tau pathology may occur via a number of pathways, including synaptic transfer, interstitial diffusion and even glial (astrocytes [109, 122], oligodendrocytes [132] and microglia [6]) intervention.

Many in vitro models have been developed to understand such uptake*.* Free aggregates present in the culture medium seem to be internalized via endocytosis [66, 75, 86, 93, 106, 143, 156, 191, 194], and this uptake might be mediated by HSPGs [93, 146, 168, 188]. The extracellular region of APP also seems to be involved in the uptake of tau fibrils in neuroblastoma cell lines [170], but other more specific receptors are also likely implicated in these mechanisms. Two entry mechanisms were recently described: the monomer of tau uses a slow actin-dependent micropinocytosis pathway, whereas aggregated tau is taken up in a rapid way independent of actin polymerization but dependent on dynamin, consistent with an endocytosis process [66]. Bin1, a neuronal amphyphisin2 isoform that is downregulated in AD brains, inhibits endocytic activity and might be involved in the propagation of tau pathology [32]. The use of double or triple microfluidic chambers is informative for studying cell-to-cell transfer as it directly links the donor to the receiving neuronal populations, emphasizing the role of synapses in cell transfer [31, 63, 143, 171, 185].

Altogether, the hierarchical pathway of neurodegeneration in the prion-like propagation hypothesis strongly suggests a highly regulated mechanism allowing specific targeting of neuronal populations. In light of the available data, this selectivity is likely to rely on receptor-mediated endocytosis.

### Tau seeding

The prion-like seeding implies that abnormal proteins can convert normal proteins into a pathological form. In this regard, Clavaguera and co-workers showed that injecting tau aggregates extracted from mice overexpressing mutated tau (P301S) into mice overexpressing human wild-type tau was sufficient to induce tau pathology [45]. When a tau-immunodepleted extract is injected, no pathology can be detected, showing that tau is the responsible factor as confirmed later by other groups [134, 179, 194]. However, this seeding activity may also require the presence of different co-factors such as polyanions [58, 201]. The same group obtained similar results by injecting human brain lysates of different tauopathies and reproducing the specific morphology of the lesions seen in the human diseases [44], supporting the concept of propagons and tau strains.

In vitro, even if a few studies showed that extracellular tau may bind to neuronal receptors (muscarinic [83], Na^+^/K^+^ ATPase (NKA) [161]…) and have toxic effects, most have shown that incubated aggregates/seeds are internalized by endocytosis and promote aggregation of overexpressed tau in cell lines [66, 75, 86, 93, 94, 134, 135, 148, 156, 170–172]. Nevertheless, most seeding assays use transfection reagents and thus bypass receptor-mediated endocytosis, which may be a critical step in prion-like propagation. In this regard, are the propagons in a free form or membrane associated? It is known that exosomes containing tau with seeding activity have been isolated from the brains of tau transgenic mice [9]. In addition, seed-competent tau species, in both free and vesicular forms, have been detected in CSF and ISF from experimental models and CSF from AD patients [85, 171]. These studies imply that tau in EVs may be endocytosed and act as a seed and therefore contribute to prion-like propagation of tau pathology. Nonetheless, as indicated above, tau in EVs is minor and immunodepleted brain extracts does not exhibit any seeding competencies.

Following the work of Clavaguera and collaborators, many subsequent studies have shown that injections of cerebral lysates or synthetic tau fibres in transgenic mice potentiate transmissibility [130]. In such experiments, human-mutated tau seeds are already overexpressed in a murine tau model, and additional seeds are injected. These works further not only support tau seeding but also indicate that it happens outside of the strict prion definition. In any case, regardless of the seeds used (artificial or brain-derived), seeding is efficient and supports this step of the prion-like propagation hypothesis [130].

Collectively, with their strengths and weaknesses, all the data generated in experimental models have greatly strengthened the prion-like propagation hypothesis. In addition, in line with the prion-like propagation hypothesis and the identification of tau in the extracellular space, free extracellular tau species are now considered key drivers in the pathology propagation by the scientific community, making them attractive targets for therapeutic approaches.

## From experimental models to clinical trials

Historically, tau therapeutic interventions were designed to target intraneuronal mechanisms such as modulating PTMs, breaking tau aggregates or decreasing tau concentrations [100]. Many of them have already failed and a global tau silencing may have side-effects due to multiple tau functions [100, 164]. More recently, tau immunotherapy showed promising preclinical results. While different mechanisms, such as microglial activation and the generation of different anti-tau antibodies, are involved in vaccination, we focus here on mechanisms involved in passive immunotherapy that have been widely explored in different experimental models [2, 7, 17, 33–35, 47, 50, 52–54, 155, 176, 178, 179, 194]. Early studies have shown that tau vaccination and tau immunotherapy could reduce intraneuronal tau pathology. Most of them decreased the amount of insoluble tau materials [7, 176, 194]. Cognitive decline was also reduced in some [176, 194], including those using MC1, an antibody recognizing a conformational epitope involving the amino terminus of tau [101] and HJ8.5, an antibody recognizing the amino acids 25–30. Both have been further developed for human immunotherapy and are now in clinical trials (LY3303560, ABBV 8E12-Table 2). Peripheral administration of this MC1 antibody in murine models of tauopathy has not only reduced tau pathology quantified by biochemical and immunohistochemical analyses but also delayed the onset of decreased motor impairment and weight loss [35]. Such experiments were also performed with a phospho-dependent antibody (anti-pS422, also further developed for human immunotherapy, RG7345-Table 2) showing similar effects with the involvement of the endosome-lysosome pathway [47]. The finding of tau secretion and its compatibility with the prion-like propagation hypothesis have modified the strategy of immune therapy: research was then oriented towards antibodies that did not penetrate the cells and principally acted in the extracellular space.

**Table 2:** Clinical trials for tau immunotherapy: to facilitate the reading, the name of antibodies currently tested in clinical trials are given, but it should be kept in mind that their murine versions have been used in experimental models to assess their mode of action

Antibodies	Isotype	Epitope	Mode of action	Target population	Clinical trial phase	Clinical Trials.gov Identifier	
BIIB076 (6C5)-Biogen	IgG1		Reduction in tau uptake and cell transfer [84, 134]	Healthy controls Mild AD	1/2	NCT03056729	
BIIB092 (IPN002)-biogen	IgG4	N-terminus	Binding eTau: reduction in neuronal activation & Aß secretion [25]	AD PSP Tauopathies	1/2	NCT03352557 NCT02460094 NCT03068468 NCT03658135	
ABBV 8E12 (HJ8.5)-abbvie	IgG4	N-terminus	Reduction in tau pathology [76, 98, 194]	PSP AD	1/2	NCT02985879 NCT03712787 NCT02880956	
JNJ-63733657-johnson & johnson		pS217	Reduction in tau seeding [150]		1	NCT03375697	
LY3303560B (MC1) lilly		N-terminus + conformation [aa7–9 and 312–34]	Reduction in tau pathology [35]	Healthy controls Mild/early AD	1/2	NCT02754830 NCT03019536 NCT03518073	
RG7345 (anti-pS422)-Roche	N/A	pS422	Reduction in tau pathology [47]	Healthy controls Discontinued development	1	NCT02281786 (RO6926496)	
RO7105705-Roche	IgG4	N-terminus	Reduction in tau pathology [8]	Healthy controls Mild AD	1/2	NCT02820896 NCT03289143 NCT03828747	
UCB0107 (antibody D)-UCb Biopharma	IgG4	Mid- region close to MTBR	Reduction in uptake, cell transfer and seeding [2, 49]	Healthy controls	1	NCT03464227	

The most advanced clinical trials (phases 1 and 2) include vaccination (AADvac1 and ACI-35, not shown) and passive immunotherapy (BIIB076, BIIB092 (Gosuranemab), ABBV-8E12 (Tilavonemab), JNJ-63733657, LY3303560 (Zagotenemab), RO7105705, UCB0107) and Lu-AF87908.

The Fc part of immunoglobulins G (IgGs) binds to specific Fc gamma receptors (FcγR), which are variously expressed in neurons, microglia or astrocytes. Different classes of FcγR have been described with high affinity (binding to monomeric IgG complexes) or low affinity (binding only to multimeric IgG complexes) to different Fc. Microglial cells highly express all classes of FcγR [4], whereas only FcγRI has been reported in astrocytes. In neurons, the expression of FcγR is still debated [136], but neurons [69] and microglia [4] seem to upregulate FcγR in response to extracellular IgG with a functional Fc domain.

The different isotypes of human IgG (IgG1, IgG2, IgG3 and IgG4) have, on the other hand, different affinities for the receptors. For example, human IgG1 has a higher affinity for activating FcγRs present on microglia compared to human IgG4 and may induce a more pro-inflammatory response [26]. IgG4 may thus theoretically bind to its antigen, lowering its toxicity, while at the same time, limiting its cell internalization through Fc receptors and the potential inflammation, it may induce [111]. Most tau antibodies currently being tested in clinical trials use this isotype to target extracellular tau (Table 2). Nevertheless, most of the preliminary studies are performed in mice, and it is often difficult to translate these data to humans. In mice, four classes are also found, but they do not correspond to human IgG. In fact, murine IgG1 is closer to human IgG4 than murine IgG2. Thus, when murine antibodies and their effector functions are tested in animal models, it is always difficult to draw conclusions. For instance, Funk and collaborators showed that the murine version of ABBV 8E12 (HJ8.5 IgG2a) drives uptake of tau species into BV2 microglial cells [76]. However, single-chain HJ8.5 antibodies without Fc fragment (scFvs) significantly reduced levels of hyperphosphorylated, aggregated tau in brain tissue of tau transgenic mice [98]. Such data suggest that the Fc fragment may not be required for the efficacy of this family of anti-tau antibodies in tau immunotherapy. The use of different approaches and different models may thus sometimes lead to different conclusions on the impact of Fc fragment and receptor.

**Fig. 6:**
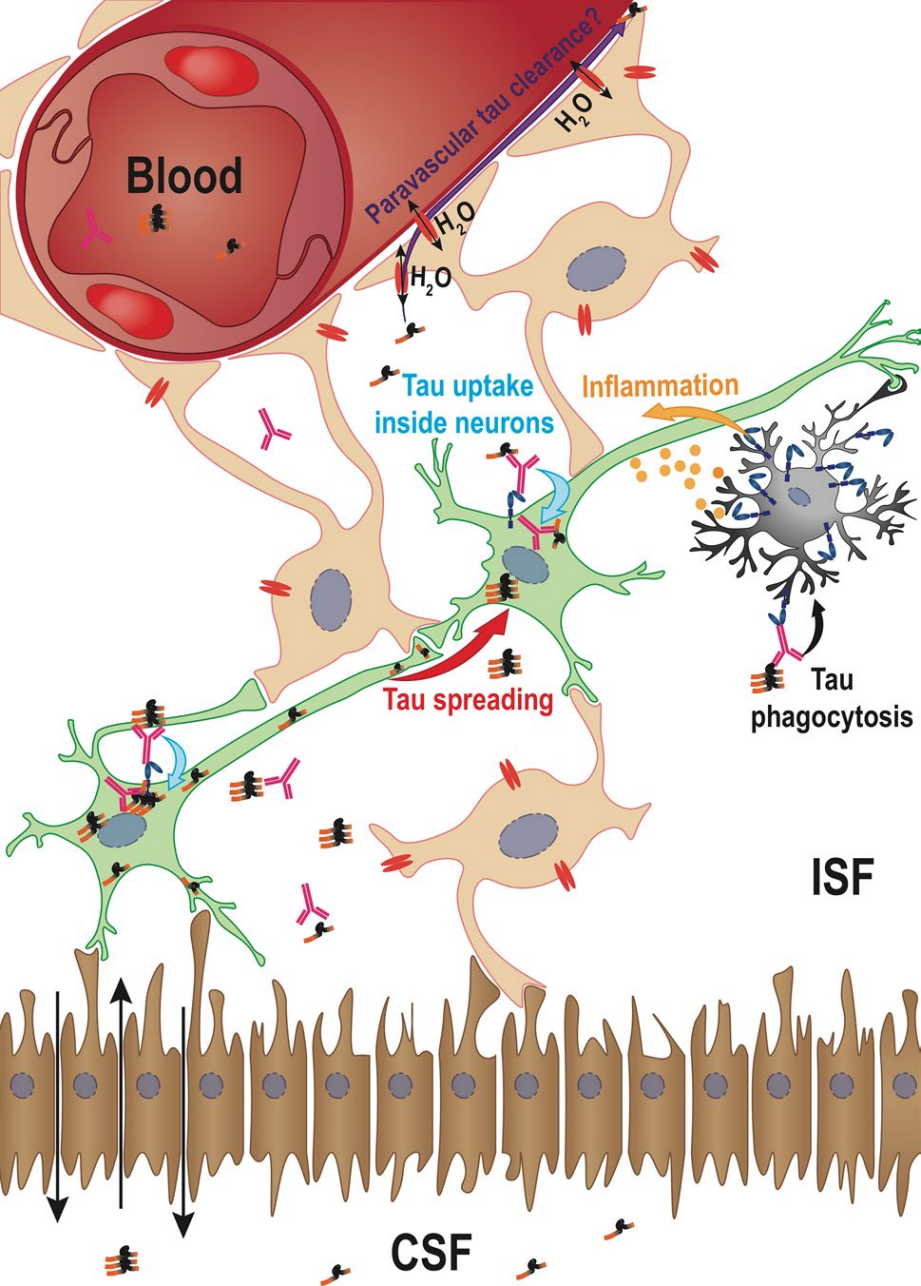
Tau clearance mechanisms-The potential for targeting extracellular tau from ISF to prevent tau pathology spreading led researchers to investigate how antibodies might be able to clear tau from the brain. Very few data are available, but among them, three major hypotheses have emerged. (1) Internalization inside neurons (blue arrow)-The first mechanism that has been described is the endocytosis of tau-Ab complexes inside neurons after binding to FcγRII/III, which might target tau to lysosomes for intracellular degradation. This degradation might amplify the propagation process by generating news seeds (red arrow). (2) Phagocytosis inside microglia (black arrow)—This second hypothesis is related to the microglial activity in the brain that might phagocytose tau and degrade it. This mode of action might be very deleterious for neighbouring neurons as the microglial phagocytosis process will generate a strong inflammatory response (yellow arrow). (3) Internalization inside astrocytes: the glymphatic tau clearance hypothesis (purple arrow). The ability of the brain to clear tau from ISF without the help of neurons and microglia is the more recent hypothesis. In this system, tau is cleared using the CSF flow through the water channel aquaporin 4 that is expressed on astrocytes and cells connected to the blood and CSF circulation via their basal ends

The most widely accepted scenario is that therapeutic antibodies bind extracellular pathological tau species in the ISF responsible for the spread of pathology but are not necessarily required to directly bind intraneuronal tau. The antibodies would block the seeding activity of extracellular tau, most likely by blocking the initial uptake of these seeds and slowing down the spread of pathology [134]. Nevertheless, the future of such anti-tau antibody-tau complexes and their clearance are still puzzling, and many hypotheses have been proposed, as summarized in Fig. 6.

Therapeutic antibodies currently in clinical development have shown their capability to target extracellular tau (by modulating cell-to-cell tau transfer) and/or to reduce tau seeding in experimental models. The murine version of BIIB076 has been shown to block neuronal uptake of human tau species in microfluidic chambers [134]. BIIB076 was described to be more efficient in blocking tau uptake than an antibody directed against the C-terminus of tau protein [134]. The murine version of UCB0107 (binding a region before the first MTBR) displayed superior efficacy in an in vitro model of seeding and aggregation induced by human AD tau than antibodies targeting N-terminal, pS202/pT205, or pS422 conformational epitopes [49]. It also effectively prevented the induction of tau pathology in the brain of transgenic mice injected with human AD brain extracts, in contrast to an amino-terminal tau antibody recognizing the same epitope as BIIB092 [2]. Other antibodies have also been tested by Vandermeeren and collaborators from Janssen [179]. Immunodepletion assays strongly suggest that difference exist between epitopes. In fact, antibodies that recognized the mild-region of tau are most effective while N-terminal antibodies could not fully block seeding in vitro (FRET assays) as well as in vivo (AD brain material enriched for PHFs injected into the hippocampus of P301L transgenic tau mouse to spur fibrillization immunized by various antibodies). Together, these results suggest that the murine version of JNJ-63733657 which is targeting the pS217, a central part of tau may also act in this way [107, 150]. Extracellular tau may also lead to neuronal hyperactivity and facilitate Aß peptide secretion; in this paradigm, it was reported that the murine version of BIIB092, IPN002, is protective by blocking extracellular tau [25].

Finally, all these antibodies have shown some effects in mouse models, although few reports are available in humans. The next challenge is to validate the target engagement and efficacy of drug candidates.

Some studies on transgenic models suggest that plasma tau is a potential biomarker for therapeutic monitoring. Indeed, the active immunization of tau transgenic mice with peptides containing a pathological phosphorylated epitope (pS422) reduced pathological tau species in the brain and delayed cognitive deficits but was importantly associated with a significant increase in plasma tau concentrations [176]. Similarly, it was reported at the 2017 AD/PD conference that injection of RO7105705 in tau transgenic mice (13 weeks treatment/3–30 mg/kg) increased plasma tau [8]. This concept of tau/Ab complex stabilization in the plasma has also been validated by another study demonstrating that peripheral administration of the anti-tau antibody HJ8.5 (ABBV 8E12) increased plasma tau not only in transgenic mice, but also in PSP patients [195]. Such stabilization in plasma may be explained by high plasma concentrations of therapeutic antibodies. For instance, at the 2017 AD/PD and AAIC conferences, it was shown that BIIB076 concentrations were 1000-fold higher in plasma than in CSF when a single injection was administered in blood in cynomolgus monkeys [84]. Tau analysis is also performed in CSF in clinical trials and may help to show target engagement. For instance, the BIIB092 antibody in a phase 1b clinical trial in PSP is well tolerated, and its complex with tau is found in CSF [18]. In fact, most of the antibodies described in Table 2 are in clinical trials, and their outcome should be published in the months to come. Nevertheless, we know that the ABBV 8E12 antibody has already been discontinued in a phase 2 clinical trial in PSP. However, it is still ongoing in AD clinical trials. BIIB092, RO7105705 and ABBV 8E12 target the amino terminal domain of tau proteins, whereas UCB0107 recognizes an epitope in the mid-region before the repeats. Only JNJ-63733657 [107] and LY3303560B [35] target so-called pathological intracellular epitopes. Knowing the molecular diversity of tau species among tauopathies, do we have the right tool for the right disease? In fact, the main question is to define which species have seeding- and propagative-competent properties.

## Tau diversity in brain and biological fluids

Since it is now acknowledged that extracellular tau (eTau) is a therapeutic target, the next generation of antibodies has to take into account the heterogeneity of tau species. This heterogeneity exists at the level of protein composition (tau sequence and PTMs), compartment (free or vesicular) and seeding competency. In fact, high heterogeneity of aggregated tau species is encountered in the human brain among tauopathies, and some of them may be released in ISF/CSF. As revealed by 2D electrophoresis studies, the tau proteome in CSF is at least as complex as that in the AD autopsy brain [88]. The pattern is composed of tau proteolytic fragments [16, 162, 199]. Barthelemy and collaborators [13, 14] identified peptides in the CSF of AD where they are abundant but also in the CSF of PSP, Lewy body dementia (LBD) and control groups where tau concentrations are very low. Among the identified peptides, those with the central part of tau (amino acids 126–234) were the most abundant ones, 4R-specific MTBR peptides were below the lower limit of detection, and the 2 N peptides were poorly detected, which might suggest that the 1N3R isoform is the most abundant isoform in CSF. When comparing pathologies, the central peptides (amino acids 126–234) were more abundant in the AD group than in the control, PSP and LBD groups. Nonetheless, no difference was observed between the PSP and LBD groups. The same observation was made by Cicognola and collaborators who described an important cleavage site at amino acid 224, especially in CSF from AD in comparison to MCI-AD, PSP and CBS [43].

The characterization of the species and nature of human tau species with seeding properties is a crucial, unanswered question. Are these seeds hypo- or hyperphosphorylated, truncated, or fibrillar? Recent studies looking precisely at seeding-competent tau from human brains with different tauopathies confirmed this molecular diversity in in vitro and in vivo models [44, 104, 105, 132, 154]. As described earlier, tauopathies are heterogeneous and there are some differences among tauopathies but we have to be aware that it is also true within a tauopathy. For instance, very little is known about the case-by-case molecular heterogeneity among a specific tauopathy such as AD and how such heterogeneity would affect the underlying pathophysiological mechanisms such as tau propagation and seeding. In this regard, Brad Hyman group described huge differences among seed-competency of human AD brain inoculates [62]. It does not mean that seeding models are bad, it only means that tau seeds in brain inoculates may be different among samples. It is not surprizing since the clinical course, the disease duration, the postmortem delay and maybe the storage conditions are different. It is important to be aware of this when seeding models are used to test and validate immunological tools. This last section highlights heterogeneity among tauopathies and strongly suggests that each disease may have its propagons. Thus, an anti-tau immunotherapy strategy common to all tauopathies is therefore unlikely.

## Conclusions

If we want to summarize our knowledge, we can say that among tauopathies, the work of neuropathologists has made it possible to define specific neurodegenerative pathways suggesting the existence of vulnerable neuronal subpopulations for each disorder. These hierarchical pathways of tau pathology led to the hypothesis of prion-like propagation, which is based on human neuropathology and numerous experimental models and has led to the identification of a new player in tauopathies: eTau. This hypothesis has led to a reorientation of anti-tau therapeutic approaches, particularly immunotherapy. The main remaining questions are around molecular species responsible for propagation or seeding and their targeting by our therapeutic strategies. Do we have the right immunological tools?

However, we should not forget that the prion-like tau propagation hypothesis is still an assumption that is not unanimous. There are first very heterogeneous tau aggregates at the molecular and cellular level among tauopathies. The diversity of neuronal populations and their connectivity are probably an explanation for differences among tauopathies. In any case, as we have seen, the arguments that support the prion-like propagation hypothesis in humans are particularly well documented in AD: the steadiness of Braak stages in AD patients and the high and constant concentration of eTau during AD pathology compared to other tauopathies. Thus, based on our current knowledge, AD is the best target for anti-eTau immunotherapy. With a remaining question, do we have the right approach since the prion-like propagation is still a hypothesis, like the amyloid cascade…
